# 3MDR Therapy Reduces Symptoms of PTSD and Related Conditions in Canadian Military Members and Veterans, Public Safety Personnel, and Clinical Personnel

**DOI:** 10.1002/brb3.71479

**Published:** 2026-05-14

**Authors:** Matthew Robert Graham Brown, Phillip R. Sevigny, Katherine S. Bright, Helen Chau, Patricia Chai, Andrew J. Greenshaw, Lisa Burback, Olga Winkler, Chelsea Jones, Suzette Brémault‐Phillips

**Affiliations:** ^1^ Department of Computing Science University of Alberta Edmonton Alberta Canada; ^2^ Heroes in Mind, Advocacy, and Research Consortium (HiMARC) University of Alberta Edmonton Alberta Canada; ^3^ Faculty of Education University of Alberta Edmonton Alberta Canada; ^4^ School of Nursing and Midwifery Mount Royal University Calgary Alberta Canada; ^5^ Faculty of Nursing University of Calgary Calgary Alberta Canada; ^6^ School of Nursing Thompson Rivers University Kamloops British Columbia Canada; ^7^ Faculty of Rehabilitation Medicine University of Alberta Edmonton Alberta Canada; ^8^ Department of Psychiatry University of Alberta Edmonton Alberta Canada; ^9^ Neuroscience and Mental Health Institute University of Alberta Edmonton Alberta Canada; ^10^ Department of Occupational Therapy, Faculty of Rehabilitation Medicine University of Alberta Edmonton Alberta Canada

**Keywords:** 3MDR therapy, first responders, military members and Veterans, PTSD

## Abstract

**Objective:**

Military Veterans, public safety personnel (police, fire fighters, paramedics, and others), and frontline healthcare workers are at elevated risk of posttraumatic stress disorder (PTSD) due to trauma exposure. Multimodal motion‐assisted memory desensitization and reconsolidation (3MDR) therapy is an innovative therapy for PTSD and trauma‐related mental health concerns, originally developed for military members and Veterans. The purpose of this study was to extend evidence for the effectiveness of 3MDR in Canadian military Veterans and to test it with public safety personnel and frontline healthcare workers.

**Methods:**

This study is a longitudinal mixed‐methods clinical trial. We examined 48 participants with PTSD from Alberta, Canada. Twenty‐two participants had treatment‐resistant PTSD. Participants were active military members or Veterans (*n* = 17), public safety personnel (*n* = 21), and/or healthcare workers (*n* = 21). Participants received a 10‐ to 14‐week course of 3MDR therapy. Quantitative data were collected pretreatment, at the end of treatment, and longitudinally at 3, 6, and 12 months after completion of 3MDR.

**Results:**

Data from 48 participants were analyzed. Data collected pre‐ and post‐3MDR therapy showed both statistically and clinically significant reductions in symptom scores for PTSD (CAPS‐5 clinical interview: 46.4 ± 2.2 to 25.1 ± 3.4, mean ± standard error; PCL‐5 questionnaire: 48.5 ± 2.2 to 32.2 ± 3.0), depression (PHQ‐9: 14.3 ± 1.0 to 9.8 ± 1.0), anxiety (GAD‐7: 12.2 ± 0.9 to 8.3 ± 0.9), and difficulties with life functioning (OQ‐45: 92.0 ± 3.9 to 77.3 ± 4.4). These improvements were maintained through to 12 months follow‐up. Resilience (CD‐RISC‐25), which was assessed pre‐ and post‐3MDR but not at follow‐up, also showed significant improvements over the course of 3MDR therapy. All results survived multiple comparison correction.

**Conclusion:**

These results support the growing body of literature illustrating 3MDR as an effective treatment for military‐related PTSD as well as PTSD‐related mental health conditions in public safety personnel and healthcare workers. These results also extend the period of post‐3MDR follow‐up to 12 months in a sample of Canadian 3MDR participants, from 6 months follow‐up in earlier Canadian 3MDR studies.

**Trial Registration:**

ISRCTN Registry 11264368; http://www.isrctn.com/ISRCTN11264368.

International Registered Report Identifier (IRRID): DERR1‐10.2196/20620

## Graphical abstract

Multi‐modal Motion‐assisted Memory Desensitization and Reconsolidation (3MDR) therapy reduces symptoms of PTSD (shown) and related mental health conditions. Figure shows mean PCL‐5 PTSD scores (and standard errors) in *n* = 48 clients with PTSD symptoms, including military members and Veterans, public safety personnel, and healthcare workers. Pre = pre‐3MDR baseline; Rec1/Rec2 = 3MDR reconsolidation sessions 1/2 at end of therapy; 3m/6m/12m = follow‐up session 3/6/12 months after 3MDR.

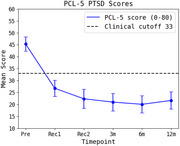

## 1 Introduction

Posttraumatic stress disorder (PTSD) and other stress‐related mental health conditions may develop from exposure to trauma (American Psychiatric Association 2013; Yehuda et al. 2015; Al Jowf et al. 2023; Burback et al. 2024). Symptoms of PTSD include negative cognitive intrusions, avoidance, hypervigilance, and alterations in mood, arousal, and reactivity (American Psychiatric Association 2013; Yehuda et al. 2015). Potentially traumatic events can involve death, threatened death, or actual or threatened serious injury to oneself or others.

Military members, public safety personnel, and healthcare workers are at risk of PTSD from operational trauma (Obuobi‐Donkor et al. 2022). Rates of PTSD among military members and Veterans have been reported at 5.3%–19% (Murphy et al. 2021; Stevelink et al. 2018; Van et al. 2016; Wagner and Jakupcak 2012; Zamorski and Boulos 2014). Among public safety personnel, including police, firefighters, paramedics, and other emergency responders, rates of PTSD have been reported at 4.7%–23.2% (Berger et al. 2012; Carleton et al. 2018, 2019; Wilson et al. 2016). Similarly, healthcare workers are at risk of PTSD from operational trauma, which was exacerbated by the COVID‐19 pandemic. Rates of PTSD among healthcare personnel during COVID‐19 were reported at 23%–40% (Andhavarapu et al. 2022; Stelnicki & Carleton 2021; Wilgenbusch et al. 2024).

Traditional therapies for PTSD show varying degrees of success. Standard treatment options for PTSD and associated mental illnesses related to military and civilian operational trauma include trauma‐focused cognitive behavioral therapy (TF‐CBT), cognitive processing therapy (CPT), prolonged exposure (PE), and eye‐movement desensitization and reprocessing (EMDR) (Department of Defence and Veterans Affairs 2017; Bisson et al. 2019; Watts et al. 2013; Burback et al. 2024). While these are evidence based and widely implemented, they may not be effective or accessible for all individuals, particularly those with complex trauma histories or treatment‐resistant symptoms (Martin et al. 2021).

### 1.1 Multimodal Motion‐Assisted Memory Desensitization and Reconsolidation (3MDR) Therapy

3MDR therapy was originally developed to treat combat‐related treatment‐resistant PTSD. 3MDR is delivered while a participant walks on a treadmill in an immersive virtual environment with a large visual display. “Multimodal” refers to the inclusion of (1) exposure to immersive visual imagery and auditory input, (2) treadmill walking, (3) a dual‐attention task, and (4) psychotherapeutic context and relationship. The theory and progression of 3MDR administration have been described elsewhere (van Gelderen et al. 2018). (Supporting Information Appendices include additional background and details of the 3MDR clinical protocol.)

Multiple studies, including four randomized controlled trials, support the effectiveness of 3MDR in military members and Veterans with treatment‐resistant PTSD at reducing PTSD, anxiety, and depressive symptoms, sustained up to 26 weeks postintervention (van Gelderen, Nijdam, Haagen, et al. 2020; van Gelderen, Nijdam, Dubbink, et al., 2020; Bisson et al. 2020; Hamilton et al. 2021; Tang et al. 2021; Jones et al. 2022; Roy et al. 2022; Smith‐MacDonald et al. 2023; Gausemel and Filkuková 2024; Nijdam et al. 2025). Nijdam et al. (2025) also included Dutch first responder participants. These findings suggest that 3MDR may offer a promising alternative or adjunct to traditional trauma‐focused therapies, particularly for individuals who have had limited symptom reduction with first‐line treatments.

We developed the Compact 3MDR System using off‐the‐shelf components, with greatly reduced financial cost and physical footprint compared to hardware in many previous studies (see Supporting Information Appendices: 3MDR Intervention). The Compact 3MDR System was used at four sites in Alberta, Canada, for this study.

Our research team conducted Phase 1 of the HiMARC 3MDR clinical trial from 2019 to 2020 with Canadian military members and Veterans with treatment‐resistant PTSD (Jones et al. 2020; Jones et al. 2022) before data collection was halted due to the COVID‐19 pandemic. The current Phase 2 of the study, started in October 2022, is a re‐commencement of 3MDR research in Alberta.

### 1.2 Study Approach and Hypotheses

This study evaluated the effectiveness of 3MDR for treating PTSD and other trauma‐related mental health conditions in several ways. (1) We continued offering 3MDR to Canadian military members and Veterans. (2) We offered 3MDR to Canadian public safety personnel (police, firefighters, paramedics, etc.) and healthcare workers with experiences of trauma, for the first time. (3) We extended the follow‐up period in a sample of Canadian 3MDR clients to 12 months, versus 6 months in earlier Canadian studies. (4) We employed the Compact 3MDR System for delivery of 3MDR. We hypothesized that mental health measures based on clinical interviews and self‐report questionnaires would improve with 3MDR treatment in military and Veteran participants, as well as public safety personnel and healthcare workers, and that improvements would endure through follow‐up 12 months later.

## 2 Materials and Methods

This study was approved by the University of Alberta's Health Research Ethics Board (Pro00084466). It received an Endorsement from the Surgeon General of Canadian Armed Forces (E2019‐02‐250‐003‐0003) authorizing recruitment of active military members.

We present an analysis of data from 48 Canadian 3MDR participants with symptoms of PTSD. Each participant belonged to one or more of three groups: military members and Veterans, public safety personnel, and/or healthcare workers. Participants completed the 3MDR therapeutic protocol and data collection pre‐ and post‐3MDR. We also present results from a subset of 30 participants who completed follow‐up data collection 3, 6, and/or 12 months after 3MDR therapy.

A convenience sample of 48 participants was recruited from the populations of regular and reserve military members and Veterans, public safety personnel (e.g., police, firefighters, paramedics), and healthcare workers in Alberta, Canada. Participants were recruited through established relationships with clinicians serving these populations, Operational Stress Injury Clinics in Alberta, the Royal Canadian Legion, other local community service providers, and self‐referral through the study website (https://3mdr.ca). All participants had clinical symptoms of PTSD based on the Clinician‐Administered PTSD Scale for DSM‐5 (CAPS‐5; Blake et al. 1995) clinical interview and/or the PTSD Checklist for DSM‐5 (PCL‐5; Weathers, Litz, et al. 2013) self‐report questionnaire. Most participants had experienced operational or occupational trauma, which was the focus of their 3MDR therapy. Some participants focused on nonoccupational traumas. Among participants actively serving as military members, public safety personnel, or healthcare workers, the trauma for which they sought 3MDR therapy interfered with their operational readiness/work availability. Many participants also had complex trauma histories. Twenty‐two participants met criteria for treatment‐resistant PTSD, meaning they had tried two or more previous therapies without success (Hamblen et al. 2019; Forbes et al. 2019). (See Supporting Information Appendices: Participant Recruitment and Inclusion Criteria.)

Participants received 3MDR therapy at the Edmonton Operational Stress Injury Clinic (EOSIC), which partnered with the research team for this study, or at one of three clinical research sites: University of Alberta main campus in Edmonton, Alberta; Alberta Hospital Edmonton; or University of Alberta space rented at the University of Calgary's downtown campus in Calgary, Alberta. (Also see Supporting Information Appendices: 3MDR Intervention.)

Consistent with a pragmatic clinical trial approach, participant allocation was guided by ethical and logistical considerations to align with real‐world treatment pathways rather than randomization to immediate treatment or waitlist control groups. When recruitment resumed in October 2022 in Phase 2 of the study, we prioritized efficiently filling clinical timeslots. Given the growing evidence for the effectiveness of 3MDR, we determined it would be unethical to withhold therapy from individuals who were in need and ready to begin treatment. We anticipated delays from scheduling variability between initial assessment and start of therapy in some participants, who would serve as a comparison group. Only two participants experienced sufficient delay (>8 weeks), so a distinct waitlist control group was not established.

The CAPS‐5 clinical interview was collected before 3MDR, at the end of 3MDR therapy (at reconsolidation session 2), and at follow‐up sessions 3, 6, and 12 months after 3MDR. Self‐report questionnaires including the PCL‐5, Patient Health Questionnaire‐9 (PHQ‐9; Kroenke et al. 2001), Generalized Anxiety Disorder Scale‐7 (GAD‐7; Spitzer et al. 2006), and other questionnaires were collected before 3MDR, during 3MDR treadmill and reconsolidation sessions, and at follow‐up sessions. (Also see Supporting Information Appendices: Data Collection.)

Statistical analyses investigated changes in CAPS‐5 scores and questionnaire scores pre‐ versus post‐3MDR therapy, as well as lack of significant changes (equivalence) from post‐3MDR through follow‐up 3, 6, and 12 months later. (For details of data processing, statistical analyses, and multiple comparison correction, see Supporting Information Appendices: Statistical Analysis.)

## 3 Results

### 3.1 Participants

From October 2022 to March 2024, 56 participants with PTSD symptoms were recruited to the study. Participants were members of one or more of three groups: military members and Veterans, public safety personnel, and healthcare workers. Our analysis included 48 participants who completed 3MDR therapy as well as pre‐ and post‐3MDR data collection. The last participants in this analysis finished 3MDR therapy in September 2024.

Eight participants were excluded from analysis. One participant withdrew from 3MDR therapy (1.7% withdrawal rate). Two participants experienced life circumstances that forced them to stop 3MDR, with the possibility of restarting in the future. Four participants completed 3MDR but opted out of post‐3MDR data collection. One participant was excluded because their baseline survey was collected after the first 3MDR treadmill session.

Of the 48 participants analyzed, 43 exhibited clinical levels of PTSD symptoms based on a CAPS‐5 score ≥30 and/or a PCL‐5 score ≥33 and/or a formal diagnosis of PTSD. Although five participants scored below the clinical thresholds, they demonstrated clinical indications of PTSD during structured clinical interviews. Their symptoms were severe enough to impair functioning across personal, social, or occupational domains, supporting their inclusion to ensure that the sample accurately reflected the range of clinically significant trauma‐related presentations encountered in practice. This approach aligns with evidence that standard self‐report measures may underrepresent clinically relevant distress in some individuals, particularly those with complex trauma histories (Herman 2015). Twenty‐two participants met criteria for treatment‐resistant PTSD, including all 17 military members and Veterans. See Table 1 for participant demographics.

**TABLE 1:** Demographics for 48 participants analyzed in this study. Some participants had multiple roles in the military, public safety, and/or healthcare.

Characteristic	Distribution
Gender	Woman: 24 Man: 24
Age (years)	Range: 30–72 Mean: 45.4 ± 10.9 (standard deviation)
Military background	Total: 17 Active Canadian military members: 2 Canadian military Veterans: 15 Regular armed forces: 7 Reserves: 2 Regular and reserves: 8 Deployed domestically: 11 Deployed internationally: 15
Public safety personnel	Total: 21 Police: 7 Fire fighter: 5 Paramedic: 4 Emergency medical services: 1 Correctional worker: 4 Private security: 1 Operational intelligence personnel: 1 Search and rescue: 4
Healthcare roles	Total: 21 Licensed practical nurse: 2 Registered nurse: 11 Nurse practitioner: 1 Psychiatric nurse: 1 Support worker: 4 Healthcare aide: 1 Physiotherapist: 1 Social worker: 1 Massage therapist: 1
COVID‐19	Public safety and/or healthcare role during COVID‐19 pandemic: 27

### 3.2 Mental Health Measures Over the Course of 3MDR Therapy

**FIGURE 1:**
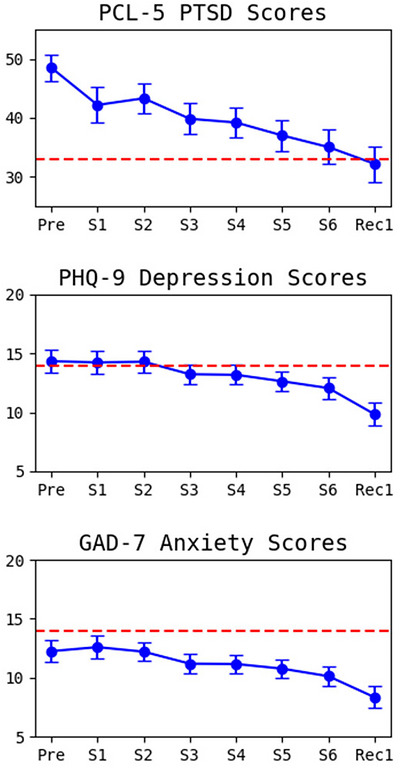
Time courses for three mental health scores during 3MDR therapy at time points: pretreatment (Pre), before each of six 3MDR treadmill sessions (S1–S6), and reconsolidation session 1 (Rec1). Data points show means across 46 participants. Error bars show standard error of the mean. Red dashed lines indicate clinical cutoffs for probable diagnoses of PTSD (PCL‐5), Major Depression (PHQ‐9), or Generalized Anxiety Disorder (GAD‐7). Decreases in scores were statistically significant for all three measures (see Table 2).

Most scores, including the PCL‐5, PHQ‐9, GAD‐7, OQ‐45, and all CAPS‐5 subscores, showed statistically significant decreases (reduction in symptoms) during 3MDR therapy that survived multiple comparison correction. CD‐RISC‐25 resilience scores showed a significant increase. AUDIT scores did not change significantly. See Table 2 and Figure 1. Each subgroup of participants (military members/Veterans, public safety personnel, healthcare workers) exhibited similar patterns of score changes during 3MDR therapy (see Supporting Information Appendices: Additional Results).

**TABLE 2:** Statistical analysis of scores pre‐3MDR (Pre) versus post‐3MDR (Post). The post‐3MDR time point is 3MDR reconsolidation session 1 for all scores except those from the CAPS‐5, for which it is 3MDR reconsolidation session 2. Pre and Post columns show mean ± standard error for each score. *N* and *p*‐value columns show numbers of participants and *p*‐values for statistical tests of changes in mean scores from pre‐ to post‐3MDR. (Also see Supporting Information Appendices: Details of Participant Numbers.) Asterisks (*) indicate *p*‐values surviving FDR multiple comparison correction (threshold *p* = 0.006). Statistical analyses used permutation tests with 100,000 iterations.

Score	Pre	Post	N	*p*‐value
PCL‐5	48.5 ± 2.2	32.2 ± 3.0	46	0.00001^*^
PHQ‐9	14.3 ± 1.0	9.8 ± 1.0	46	0.00001^*^
GAD‐7	12.2 ± 0.9	8.3 ± 0.9	46	0.00001^*^
OQ‐45	92.0 ± 3.9	77.3 ± 4.4	45	0.00002^*^
AUDIT	3.8 ± 0.6	3.5 ± 0.7	44	0.4
CD‐RISC‐25	58.0 ± 2.2	65.3 ± 2.5	44	0.0007^*^
CAPS‐5 Total Symptom Score	46.4 ± 2.2	25.1 ± 3.4	30	0.00002^*^
CAPS‐5 B Re‐experiencing	12.1 ± 0.7	5.8 ± 0.9	30	0.00001^*^
CAPS‐5 C Avoidance	5.9 ± 0.3	2.6 ± 0.5	30	0.00001^*^
CAPS‐5 D Neg Alterations	15.6 ± 0.9	8.8 ± 1.3	30	0.00001^*^
CAPS‐5 E Hyperarousal	12.8 ± 0.8	7.9 ± 0.9	30	0.00006^*^
CAPS‐5 Dissociation	3.4 ± 0.5	1.0 ± 0.3	30	0.00005^*^

### 3.3 Mental Health Outcomes 12‐Months Post‐3MDR Therapy

**FIGURE 2:**
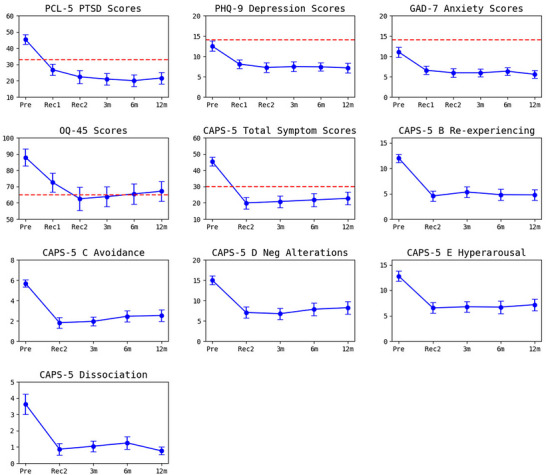
Time courses up to 12 months after 3MDR therapy for time points: pretreatment (Pre), 3MDR reconsolidation sessions 1 and 2 (Rec1, Rec2), and 3, 6, and 12 months follow‐up (3m, 6m, 12m). Data points show means across 23 participants (PCL‐5, PHQ‐9, GAD‐7, OQ‐45) or 22 participants (CAPS‐5 subscales). Error bars show standard error of the mean. Red dashed lines indicate the clinical cutoffs for probable diagnoses of PTSD (PCL‐5, CAPS‐5 Total Symptom Score), Major Depression (PHQ‐9), Generalized Anxiety Disorder (GAD‐7), or a clinical concern with life functioning (OQ‐45). Decreases in scores for all measures were statistically significant from the Pre to Rec2 (see Table 3). Decreases in scores at Rec2 were maintained over follow‐up at 3, 6, and 12 months (see Table 3).

In a subset of participants (*n* = 30), data were collected at follow‐up sessions 3, 6, and/or 12 months after 3MDR therapy (18 participants did not respond to contact attempts for follow‐up). As shown in Table 3 and Figure 2, all scores showed a statistically significant decrease (improvement) from pre‐ to post‐3MDR therapy. Decreases were maintained, with scores showing a statistically significant equivalence from the end of therapy through 3, 6, and 12‐month follow‐up. (AUDIT and CD‐RISC‐25 were not collected at follow‐up.) Each subgroup of participants (military members/Veterans, public safety personnel, healthcare workers) showed a similar pattern of changes (see Supporting Information Appendices: Additional Results).

**TABLE 3:** Statistical analysis of scores collected at pre‐3MDR baseline, reconsolidation, and follow‐up for the subset of 30 participants who completed data collection at Pre, Rec1/Rec2, and one or more follow‐up time points. Time points include pre‐3MDR baseline (Pre), 3MDR reconsolidation sessions 1 and 2 (Rec1, Rec2), and follow‐up at 3, 6, and 12 months (3m, 6m, 12m). The Pre, Rec1, Rec2, 3m, 6m, and 12m columns show means ± standard errors. CAPS‐5 measures were not collected at Rec1. *N* column shows the number of participants. (Also see Supporting Information Appendices: Details of Participant Numbers.) “P Pre‐Post” column shows *p*‐values for statistical tests of changes in mean scores over the Pre, Rec1, and Rec2 time points. “P Equiv” column shows *p*‐values for statistical equivalence tests for a lack of increase over the Rec1, Rec2, and follow‐up 3m, 6m, and 12m time points. Asterisks (*) indicate *p*‐values surviving FDR multiple comparison correction (threshold *p* = 0.006). Statistical analyses used permutation tests or bootstrap tests with 100,000 iterations.

Score	Pre	Rec1	Rec2	3m	6m	12m	*N*	P Pre‐Post	P Equiv
PCL‐5	45.3 ± 3.0	26.8 ± 3.4	22.4 ± 3.9	21.0 ± 3.7	20.1 ± 3.6	21.7 ± 3.6	29	0.00001^*^	0.00001^*^
PHQ‐9	12.5 ± 1.3	8.1 ± 1.1	7.3 ± 1.2	7.5 ± 1.2	7.4 ± 1.0	7.2 ± 1.1	29	0.00001^*^	0.00001^*^
GAD‐7	11.0 ± 1.3	6.6 ± 1.0	6.0 ± 1.0	6.0 ± 1.0	6.3 ± 1.0	5.6 ± 0.9	29	0.00001^*^	0.00001^*^
OQ‐45	87.9 ± 5.2	72.6 ± 5.8	62.5 ± 7.1	63.8 ± 6.1	65.5 ± 6.2	67.1 ± 6.1	29	0.00001^*^	0.00001^*^
CAPS‐5 Total Symptom Score	45.4 ± 2.6	—	20.0 ± 3.6	20.8 ± 3.6	21.8 ± 4.1	22.7 ± 3.8	22	0.00002^*^	0.00001^*^
CAPS‐5 B Re‐experiencing	12.0 ± 0.8	—	4.5 ± 1.0	5.3 ± 1.1	4.8 ± 1.1	4.8 ± 1.0	22	0.00002^*^	0.00001^*^
CAPS‐5 C Avoidance	5.7 ± 0.4	—	1.8 ± 0.5	2.0 ± 0.4	2.5 ± 0.5	2.5 ± 0.6	22	0.00001^*^	0.006^*^
CAPS‐5 D Neg Alterations	15.0 ± 1.0	—	7.0 ± 1.4	6.8 ± 1.4	7.8 ± 1.6	8.2 ± 1.5	22	0.00006^*^	0.00001^*^
CAPS‐5 E Hyperarousal	12.8 ± 1.0	—	6.5 ± 1.0	6.8 ± 1.0	6.7 ± 1.2	7.2 ± 1.1	22	0.0002^*^	0.00001^*^
CAPS‐5 Dissociation	3.6 ± 0.6	—	0.9 ± 0.4	1.0 ± 0.3	1.2 ± 0.4	0.8 ± 0.2	22	0.0007^*^	0.00001^*^

## 4 Discussion

This study examined the effectiveness of 3MDR therapy in treating PTSD and other mental health concerns related primarily to operational trauma among a diverse cohort of 48 Canadian participants including military members and Veterans, public safety personnel, and healthcare workers. Statistically and clinically significant reductions were observed in symptoms of PTSD, major depressive disorder, and generalized anxiety disorder. Participants also reported meaningful improvements in overall life functioning and psychological resilience. Notably, these therapeutic gains were maintained at the 12‐month follow‐up, suggesting sustained benefits of 3MDR over time.

### 4.1 Military Combat‐Related PTSD

The results add to the growing body of evidence for the effectiveness of 3MDR in treating combat‐related, treatment‐resistant PTSD. Randomized control trials from the Netherlands, the United Kingdom, and the United States have all shown a statistically significant reduction in CAPS‐5, PCL‐5, and GAD‐7 scores (van Gelderen, Nijdam, Haagen, et al. 2020; Bisson et al. 2020; Roy et al. 2022, Nijdam et al. 2025), as have our previous studies of 3MDR in Alberta, Canada (Jones et al. 2022).

### 4.2 Trauma in Public Safety Personnel and Healthcare Workers

We applied 3MDR therapy for the first time in a group of Canadian public safety personnel (police, firefighters, paramedics, etc.) and healthcare workers to treat PTSD and related mental health concerns caused by trauma. These participants reported one or more index traumas in the form of operational, occupational, or other traumas. These traumas interfered with their ability to serve in their professional roles. Many also had histories of complex trauma stemming from earlier life events, and these additional experiences often compounded the impact of their index trauma and contributed to the severity or complexity of their clinical presentation. On average, these participants showed significant improvements in scores for PTSD, major depressive disorder, generalized anxiety disorder, life functioning, and resiliency during the course of 3MDR therapy. Improvements were still present at follow‐up 12 months after 3MDR therapy concluded. These results provide initial support for 3MDR as an effective therapy for PTSD and other mental health concerns related to trauma in civilian populations.

### 4.3 Limitations

A pragmatic trial design was employed to reflect real‐world clinical conditions, and, therefore, participant allocation did not include randomization to a waitlist control group, as explained previously. We expect that future studies will include participants naturally randomized to a waitlist control group due to scheduling variations.

CAPS‐5 data were lost for some participants. The CAPS‐5 data that are available support the effectiveness of 3MDR therapy, as do the PCL‐5 questionnaire PTSD score data.

The subset of 30 participants who completed follow‐up data collection was weighted unequally among the three participant groups, with four military members/Veterans, eight public safety personnel, and 19 healthcare workers (see Supporting Information Appendices). Patterns of score changes were similar among all three subgroups, but the evidence at follow‐up is most strongly weighted toward healthcare workers.

Trauma interventions beyond the 3MDR therapy were controlled for during the administration of 3MDR therapy but not after 3MDR had concluded. In addition, there was variation in participants’ life events, circumstances, and levels of support outside of the 3MDR intervention. The outcomes described here could have been influenced by factors beyond 3MDR therapy not considered in the analysis.

Comparing participants with versus without treatment‐resistant PTSD would have been informative but was confounded by participant subgroup membership. The treatment‐resistant group (*n* = 22) was composed mostly of the military members/Veterans subgroup (*n* = 17).

## 5 Conclusions

The results from this clinical study support the effectiveness of 3MDR as a treatment for combat‐related, treatment‐resistant PTSD in military members and Veterans, as well as for PTSD related to operational trauma in public safety personnel and healthcare workers. We observed statistically significant improvements in symptoms of PTSD, generalized anxiety disorder, major depressive disorder, as well as life functioning and resiliency after 3MDR and at 12 months posttreatment. This study adds to a growing literature supporting 3MDR as a therapy for treatment‐resistant PTSD in military members and Veterans, and it extends 3MDR to treatment of PTSD in civilian participants including public safety personnel and healthcare workers. The study extends the follow‐up period in a sample of Canadian 3MDR clients from 6 months reported previously (Jones et al. 2022) to 12 months. Finally, the study supports the effectiveness of the Compact 3MDR System hardware for use in 3MDR therapy.

## Author Contributions

**Matthew Robert Graham Brown**: conceptualization, investigation, funding acquisition, writing – original draft, methodology, validation, visualization, writing – review and editing, software, formal analysis, project administration, supervision, data curation, resources. **Phillip R. Sevigny**: conceptualization, investigation, funding acquisition, writing – original draft, methodology, validation, writing – review and editing, project administration, supervision, data curation, resources. **Katherine S. Bright**: conceptualization, investigation, funding acquisition, methodology, validation, writing – review and editing, project administration, supervision, data curation, resources. **Helen Chau**: investigation, validation, writing – review and editing, data curation, resources. **Patricia Chai**: investigation, validation, writing – review and editing, data curation, resources. **Andrew J. Greenshaw**: conceptualization, funding acquisition, writing – review and editing. **Lisa Burback**: conceptualization, investigation, funding acquisition, methodology, validation, writing – review and editing, project administration, supervision, data curation, resources. **Olga Winkler**: conceptualization, investigation, funding acquisition, methodology, validation, writing – review and editing, project administration, supervision, data curation, resources. **Chelsea Jones**: conceptualization, investigation, funding acquisition, methodology, validation, writing – review and editing, project administration, supervision, data curation, resources. **Suzette Brémault‐Phillips**: conceptualization, investigation, funding acquisition, methodology, validation, writing – review and editing, project administration, supervision, data curation, resources.

## Conflicts of Interest

The authors declare no conflicts of interest.

## Supporting information




**Supplementary Materials**: brb371479‐sup‐0001‐SuppMat.docx

## Data Availability

Due to privacy/ethical restrictions, the data used in this study are available only on request from the corresponding author, SBP. The data are not publicly available due to their containing information that could compromise the privacy of research participants.
