# Supplementary material for: 3MDR Therapy Reduces Symptoms of PTSD and Related Conditions in Canadian Military Members and Veterans, Public Safety Personnel, and Clinical Personnel
Source: Brain Behav. 2026 May 14;16(5):e71479. doi: 10.1002/brb3.71479 (PMC13175268; doi:10.1002/brb3.71479)
Supplement: Supplementary file 1 — Supplementary Materials: brb371479‐sup‐0001‐SuppMat.docx [file BRB3-16-e71479-s001.docx]

**Appendix 1 - Overview of Multi-modal Motion-assisted Memory Desensitization and Reconsolidation (3MDR) Therapy**

Multi-modal Motion-assisted Memory Desensitization and Reconsolidation (3MDR) therapy is an emerging treatment for PTSD and potentially other trauma-related mental health concerns. 3MDR is delivered while a participant walks on a treadmill in an immersive virtual environment that includes a large visual display. A course of 3MDR therapy involves a series of weekly sessions including 1-3 initial preparatory sessions (2 preparatory sessions in the current study), a course of 6-10 3MDR treadmill sessions, and two 3MDR reconsolidation sessions.

The preparatory sessions focus on building rapport, creating a sense of safety, and orienting the participant to the 3MDR process. Therapists gather a detailed history, clarify goals, and identify the index trauma to be worked on during 3MDR while introducing grounding and self-regulation skills to support emotional stability. Participants also select meaningful images and music to use in their treadmill sessions. Finally, they are familiarized with the 3MDR equipment and process using neutral or positive images and music so that they are comfortable progressing to trauma processing.

3MDR treadmill sessions involve the client walking on the treadmill, listening to music at the beginning and end, and viewing a series of 7 client-specific images embedded in the 3MDR visual display, while receiving psychotherapy from the 3MDR clinician, further described below. The 3MDR treadmill sessions take place in an immersive environment including a treadmill and full field visual display. Each treadmill session lasts 90 minutes and includes three phases: (A) a *pre-platform phase*; (B) a *platform phase*, and (C) a *post-platform phase*. During the *pre-platform phase*, the clinician and participant first choose and order 7 personalized images reflective of the participant’s trauma, and select two pieces of music for use in the session. In the *platform phase*, which takes 45-60 minutes, the participant first engages in a brief warm-up during which they walk on the treadmill while listening to self-selected music with a clinician standing alongside. This is followed by a series of seven 4-5 minute sequential cycles of brief exposure-based psychotherapy. Guided by targeted questions from the clinician, participants “touch into and out of” their trauma, as they engage in narrative, emotional, and somatosensory processing. A subset of selected participant words (i.e., labels) are displayed in the foreground of the image. After reading aloud the labels, the participant engages in a brief 60 second EMDR-based dual attention task, during which they read aloud numbers displayed on a red ball moving back and forth horizontally across the image. Subsequent cycles follow using each of the self-selected images. After the seventh image, the participant enters a cool-down period of walking while listening to self-selected cool-down music. Once off the treadmill, the clinician and participant engage in a 15-20 minute *post-platform phase* of reconsolidation, consisting of talk therapy and meaning making of the previous hour of 3MDR therapy. This includes a review of the session, creation of space for more adaptive thoughts and emotions, discussion of new insights, and review of a self-care plan.

Reconsolidation sessions (last two clinical 3MDR sessions) follow completion of all 6-10 treadmill sessions to facilitate meaning making and integration of the therapeutic experience. Each reconsolidation session is a 60-90 minute session of psychotherapy and meaning making over the entire 3MDR therapeutic experience. During each of these two sessions, the participant’s overall experience of the therapy is discussed, including any effects it may have had on various aspects of their lives, relationships, social, occupational, and functioning. Their perspectives on 3MDR are also explored.

**Appendix 2 - Additional Details of Materials and Methods**

**Participant Recruitment and Inclusion Criteria**

Participants were eligible for the study if they had experienced operational trauma, occupational trauma, or other trauma affecting their job performance; were English-speaking; aged 18+; had the capacity to walk on a treadmill for at least 45 minutes; and met one or more of the following criteria:

- A diagnosis of PTSD based on the Diagnostic and Statistical Manual-5 (DSM-5), with symptoms lasting more than 3 months;

- A score of 30 or higher on the Clinician-Administered PTSD Scale, Fifth Edition (CAPS-5) clinical interview with symptoms lasting more than 3 months;

- A score of 33 or higher on the PCL-5 self-report questionnaire on PTSD with symptoms lasting more than 3 months;

- A history of trauma-related mental health symptoms lasting more than 3 months and with severity sufficient to impact personal, social, or and/or occupational aspects of the participant’s life, based on a clinical assessment by the prospective 3MDR therapist.

22 participants also met criteria for treatment-resistant PTSD, in that they had not responded to at least two previous evidence-based PTSD treatments, at least one being a psychotherapeutic intervention and the second treatment being a pharmacological or psychotherapeutic intervention.

Participants were required to be stable on any psychotropic medication for a period of 4 weeks before entering the study. Those with comorbidities were included if they satisfied the other criteria and PTSD symptoms were considered the primary clinical concern.

Potential participants were either existing participants known to a given clinical group collaborating with the study, or they were potential new participants, not yet known to the group or to the research team. In the case of an existing participant known to a clinical group, if that individual met the study inclusion criteria and if their primary clinician considered 3MDR to be a good option for them, then their clinician offered them the opportunity to receive 3MDR therapy as part of the research study. If the individual agreed, they provided verbal and written consent and entered the study as a participant. Importantly, in such cases, the clinician always had a primary appointment at the collaborating mental health clinic, with either no formal affiliation with the research team or else only a secondary affiliation with the research team. The clinician's primary responsibility was always to the participant, not to the research study, to safeguard participant safety and voluntary participation.

Potential participants not previously known to the research team or to collaborating clinics were referred to the study either by self referral (contact form on the study website) or by a clinician not involved with the study. Such individuals were screened by a member of the research team to discuss their military employment/deployments if applicable, history in a public safety personnel and/or healthcare role if applicable, history of operational trauma, current and past medical history, history and experiences of previous PTSD interventions, and overall suitability prior to providing verbal and written consent to participate in the study.

**3MDR Intervention**

The 3MDR clinical protocol involved a 10-14 week program, with one session per week, including two 3MDR preparatory sessions, 6-10 3MDR treadmill intervention sessions, and two 3MDR clinical reconsolidation sessions. In this study, participants received 6 3MDR treadmill sessions, with the option for up to 4 additional treadmill sessions (10 total) at the discretion of the 3MDR clinician. The last two sessions, 1 week and 2 weeks after the last treadmill session, were 3MDR clinical reconsolidation sessions, which involved meaning-making of the previous weeks of 3MDR therapy, in the context of a psychotherapeutic session. In addition to being clinical sessions, the reconsolidation sessions also included data collection with self-report surveys and the CAPS-5 clinical interview at reconsolidation session #2. Participants were contacted again at 1-, 3-, 6-, 12-, 24- and 36-months post intervention for follow-up sessions and data collection.

Many previous 3MDR studies used the large, expensive Computer Assisted Rehabilitation ENvironment (CAREN) or Gait Real-time Interactive Laboratory (GRAIL) from Motek Medical BV, Netherlands (Bisson et al. 2020; Hamilton et al. 2021; Jones et al. 2022; Roy et al. 2022; Smith-MacDonald et al. 2023; Tang et al. 2021; van Gelderen et al. 2020a, 2020b). For the 3MDR treadmill sessions, the current study used the Compact 3MDR System hardware designed by our research group. The Compact 3MDR System design includes three large format televisions, a treadmill, fall protection setup, and 3MDR control computer. Each television has a diagonal length of 85 inches, with widescreen format (16:9 width:height ratio). The virtual scene is blended across the three screens. The treadmill is an HP Cosmos T150 DE MED, a large and sturdy treadmill with integrated fall protection system. The treadmill is oriented backwards, pointing the centre control console away from the screens and allowing the participant an unimpeded view of the screen array. Version 2.2.0 of the 3MDR Software (Naval Health Research Center, San Diego, CA) was used for this study. The treadmill was controlled separately from the 3MDR software.

Of the 48 participants in this study, 44 completed the 10-14 week 3MDR therapeutic program described above, while four participants had protocol deviations (detailed below). One participant completed 4 treadmill sessions; 2 participants, 5 treadmill sessions; 24 participants, 6 treadmill sessions; 2 participants, 7 sessions; 4 participants, 8 sessions; 3 participants, 9 sessions; and 12 participants, 10 sessions. In some cases, the timing of sessions was altered to accommodate participant or clinician scheduling, for example skipping one week (i.e. pushing back the remaining timeline by one week) due to lack of availability.

One participant completed four 3MDR treadmill sessions, responding well during that time, as well as 3MDR reconsolidation sessions and follow-up. This participant reported feeling that they were able to move forward after only four treadmill sessions and opted out of treadmill sessions 5 and 6.

Two participants only completed five 3MDR treadmill sessions. One responded very well after five treadmill sessions as per their own assessment and that of their 3MDR clinician, and they did not do a sixth treadmill session. One participant did not exhibit significant changes in mental health scores after 5 treadmill sessions and did not complete a 6th treadmill session, though they did complete post-3MDR data collection.

One participant who completed ten 3MDR treadmill sessions also received two extra non-treadmill therapeutic sessions after treadmill sessions #6 and #8 as they were not in a state to do 3MDR treadmill sessions on those days.

**Data Collection**

Standardized self-report clinical outcome measures were filled out by participants via a secure online survey system at various time points. Clinical interview data were also collected at various times. A large self-report questionnaire battery was collected at baseline before 3MDR therapy commenced (Pre time point). The baseline battery was collected either as part of a separate assessment and screening session for potential participants or as part of 3MDR preparatory session 1, when a given participant was already known to the clinical group. The baseline battery included the questionnaires listed below as well as a demographics questionnaire. A large questionnaire battery was also collected at 3MDR reconsolidation session 1 (Rec1), including all instruments from the baseline battery except for the demographics and Life Events Checklist-5 (LEC-5) questionnaires to reduce time requirements. Shorter questionnaire batteries were collected at the start of each 3MDR treadmill session (S1, S2, ...), at 3MDR reconsolidation session 2 (Rec2), and at follow-up sessions 3, 6, and 12 months (3m, 6m, 12m) after reconsolidation session 2. The self-report and clinician administered instruments are detailed below, including which instruments were included at which time points.

1. The self-report version of the Life Events Checklist for DSM-5 (LEC-5, Weathers et al. 2013a, b) was collected as part of the baseline (Pre) questionnaire battery. The LEC-5 provides an overview of a participant's prior trauma experiences.
2. The PTSD Checklist for DSM-5 (PCL-5) (Weathers et al. 2013b): The PCL-5 assesses PTSD symptom severity on a scale from 0 to 80. The PCL-5 is a 20-item self-report questionnaire on symptoms in relation to an identified “stressful experience”, with each symptom rated on a 0-4 scale. We collected the PCL-5 at time points Pre, S1, S2, ..., Rec1, Rec2, 3m, 6m, and 12m.
3. The Patient Health Questionnaire-9 (PHQ-9) (Kroenke et al. 2001): The PHQ-9 assesses the severity of depression. The PHQ-9 incorporates DSM-IV depression diagnostic criteria into a 9-item, self-report questionnaire. Responses represent the frequency of symptoms in the past two weeks, with each symptom rated on a 0-3 scale (Kroenke et al. 2001). A score of 5-9 indicates mild depression; 10-14 indicates moderate depression; 15-19 indicates moderately severe depression; and 20-27 indicates severe depression. We collected the PHQ-9 at time points Pre, S1, S2, ..., Rec1, Rec2, 3m, 6m, and 12m.
4. The Generalized Anxiety Disorder Scale-7 (GAD-7) (Spitzer et al. 2006): The GAD-7 measures the severity of anxiety. This self-report questionnaire includes 7 items asking about the frequency of symptoms in the two past weeks on a 0-3 scale. A score of 5-9 indicates mild anxiety; 10-14 indicates moderate anxiety; and 15-21 indicates severe anxiety. We collected the GAD-7 at time points Pre, S1, S2, ..., Rec1, Rec2, 3m, 6m, and 12m.
5. The Outcomes Questionnaire-45 (OQ-45) (Lambert et al. 1996): The OQ-45 is a self-report questionnaire assessing social functioning. It includes 45 items, with an overall score range of 0-80. A total score of 63 is clinically significant, and a change of 14 points or more between sessions is clinically significant. We collected the OQ-45 at time points Pre, Rec1, Rec2, 3m, 6m, and 12m.

1. The Alcohol Use Disorder Identification Test (AUDIT) (Bradley et al. 2003): The AUDIT is an alcohol self-report questionnaire with 10 items that assesses hazardous drinking or active alcohol use disorder. A score of 8 or more indicates hazardous or harmful alcohol use. We collected the AUDIT at time points Pre and Rec1
2. The Connor Davidson Resilience Scale (CD-RISC) (Connor and Davidson 2003): The CD-RISC self-report questionnaire assesses resilience using 25 questions. We collected the CD-RISC-25 at time points Pre and Rec1.

Other questionnaires were also collected in addition to those listed above, but these will be reported in separate publications.

The DSM-5 Clinician-Administered PTSD Scale, Fifth Edition (CAPS-5) (Blake et al. 1995): The CAPS-5 is a 29-item structured clinical interview for assessing PTSD diagnostic status and symptom severity (Blake et al., 1995). The CAPS-5 is meant to be informed by the LEC-5, which we collected in the baseline (Pre) questionnaire battery. The CAPS-5 is the gold standard in PTSD assessment. The past month and worst month versions of the CAPS-5 provide a current (past month) diagnosis of PTSD. The past week version of the CAPS-5 assesses symptoms over the past week. The CAPS-5 past month version was collected at baseline (Pre), either on the same day as the baseline questionnaire battery or a different day close to the baseline battery, as determined by each participant's schedule. The CAPS-5 past month version was also collected at follow-up at 3m, 6m, and 12m. The CAPS-5 last week version was collected at reconsolidation session 2 (Rec2). In the analysis, we considered the CAPS-5 Total Symptom Score as well as five CAPS-5 subscores: CAPS-5 B Re-experiencing, CAPS-5 C Avoidance, CAPS-5 D Negative Alterations, CAPS-5 E Hyperarousal, and CAPS-5 Dissociation.

The CAPS-5 interview was collected by the participant's 3MDR clinician, using the standard procedure which focuses on an index trauma. In most cases, the clinicians recorded interview data on paper sheets, these were handed off to the research team, and the data were digitized into a secure online system to allow for later analysis. In some cases, clinicians directly entered the CAPS-5 data into the digital system during the interview.

Originally, post-3MDR data collection was scheduled to occur one week after the final 3MDR clinical session (3MDR Reconsolidation Session 2, or Rec2). However, scheduling and logistical challenges led to incomplete follow-up data collection for several participants, resulting in some data loss. To address this, we revised our protocol: the full post-3MDR questionnaire battery was administered during 3MDR Reconsolidation Session 1 (Rec1), while the post-3MDR CAPS-5 interview (past-week version) was conducted at Rec2. A shorter questionnaire battery was also administered at Rec2, as previously described. This adjustment significantly improved data completion rates at the conclusion of 3MDR therapy.

**Statistical Analysis**

As required by the University of Alberta's Health Research Ethics Board, participants had the option to not fill in all the questions in the self-report questionnaires. Mean imputation was used to fill in missing values, though most participants did answer all the questions. Specifically, missing answers were filled in with mean imputation as long as at least 75% of the answers were provided for a given questionnaire. If a participant provided fewer than 75% of the answers needed for a questionnaire score, that questionnaire score was treated as missing for that participant for that time point. Considering all score values computed for all participants, questionnaires, and time points, 99.89% of all score values were computed from full data, 0.11% of score values were computed with 75% or more of the underlying answers available (using mean imputation for missing answers), and no score values were treated as missing because less than 75% of the underlying answers were available. Therefore, in a very small proportion of questionnaire scores, some scores were missing for specific participants, for specific time points. Missing data could also occur for a specific participant and time point because that participant did not complete a questionnaire battery or a CAPS-5 interview for that time point. This pertained primarily to the follow-up time points, where a participant may have completed some but not all follow-up time points (3, 6, 12 months). Handling of missing data is described below.

Statistical testing to assess changes in mental health scores over time was done using linear models and permutation testing. For a given score and set of two or more time points, the dependent values were the scores for each participant at those time points. The linear model included a linear term to model changes over time (two or more time points) and one offset term per participant to account for between-subject variability. The model was fit to the data using least-squares. The fitted slope parameter from the linear term was taken as a measure of the score’s change over time. Where necessary, the linear model was adjusted to handle missing data by removing the missing time points from the dependent score values and from the linear model. We used permutation testing to test the estimated slope parameter for statistical significance, for each score and set of time points. Permutation testing was chosen because it is a non-parametric method that does not require assumptions about the shape of the statistical distribution of the data (including no assumption that the data are normally distributed). For each score, we generated an empirical distribution of the estimated linear slope parameter including 100,0000 samples. 99,999 iterations of permutation were used to generate all but one of the samples, and the actual estimated slope parameter comprised the last sample, as is standard practice. On each iteration, the time point labels were permuted randomly and separately within each participant, and the linear model was then fit to the permuted data to generate an estimated slope parameter. Where missing data points were present, permutation did not interact with the missing data. (That is, a missing data point was never exchanged with a valid data point during permutation.) The actual estimated slope parameter was then compared against the empirical distribution to generate a p-value.

Statistical equivalence testing was done to test for a significant lack of increase in a score over a set of time points. The same linear model procedure was used as described above to generate a slope parameter for a given set of data at two or more time points. Bootstrap testing was then used to compare the observed slope value to a reference value. An empirical distribution of the slope parameter was created, comprised of 100,000 samples. Over each of 99,999 iterations, a new data sample was generated by sampling from the set of participants with replacement, and including all data points from each sampled participant (with possibility of multiple copies of a participant's data being included, due to replacement). The linear model was fit to the resampled data and the slope parameter extracted. The model was then fit to the actual data and the slope parameter extracted and added as the final sample in the empirical distribution. The empirical distribution of slope values was then compared against a reference slope value defined such that slope values below the reference were considered "small" or equivalent to zero. That is, a p-value was generated as the proportion of empirical distribution values equal or greater than the reference value. (See below for further details of reference values for specific scores.)

The following 12 tests were done to assess changes during the course of 3MDR therapy. For the PCL-5, PHQ-9, and GAD-7 scores, we tested changes over time points Pre, S1, S2, S3, S4, S5, S6, and Rec1. For the OQ-45, AUDIT, and CD-RISC-25 scores, we tested changes from Pre to Rec1. For the CAPS-5 Total Symptom Score and five CAPS-5 subscores, we tested changes from Pre to Rec2, as CAPS-5 was collected at Rec2 not Rec1.

All participants were re-contacted for follow-up sessions at 3, 6, and 12 months post 3MDR. 18 of 48 participants did not respond at any follow-up time points. The remaining subset of n=30 participants completed follow-up data collection 3, 6, and/or 12 months after reconsolidation session 2. This subset of participants was analyzed to test for persistence of changes during 3MDR therapy over the course of follow-up 12 months after the end of 3MDR. Firstly, the following 10 tests were done to assess changes from pre- to post-3MDR therapy in this subset of participants. For the PCL-5, PHQ-9, GAD-7, OQ-45, we tested changes from Pre through Rec1 and Rec2, using permutation testing with the method described above. CAPS-5 interviews were collected only at Rec2, not Rec1. For the CAPS-5 Total Symptom Score and five CAPS-5 subscores, we tested changes from Pre to Rec2. In addition, 10 equivalence tests were done to assess a statistically significant lack of increase from the end of 3MDR through follow-up at 3, 6, and 12 months for the same set of 10 scores. “End of 3MDR” was Rec1 for questionnaire scores (PCL-5, PHQ-9, GAD-7, OQ-45) and Rec2 for CAPS-5 scores. The reference slope value for the equivalence test was defined as an increase of one quarter of the total score range over the five time points Rec1 to 12m for questionnaire scores or one quarter of the total score range over the four time points Rec2 to 12m for CAPS-5 scores. (Note that the AUDIT and CD-RISC-25 were not collected at Rec2 nor at follow-up time points.)

We performed a total of 32 statistical tests using data from the entire set of n=48 participants, and we used the Benjamini-Hochberg procedure for false discovery rate (FDR) correction (Benjamini and Hochberg 1995) to address multiple comparisons over all 32 tests. Results from these analyses are presented in the main text.

The 48 participants fell into three subgroups including military members and Veterans (n=17), public safety personnel (n=21), and healthcare workers (n=21). A given participant could belong to multiple subgroups. To investigate whether results from the set of 48 participants might be driven more or less by one or two subgroups, we also performed analyses on each of the subgroups of participants. These analyses were the same as described above for the entire set of participants, but limited to participants from a given subgroup. Multiple comparison correction was not done for the subgroup analyses because the subgroup analyses were repeats of the aggregate analyses using the same data subdivided differently. The results of participant subgroup analyses are included below, in subsection Results for Analyses of Participant Subgroups.

Follow-up data were collected 3, 6, and/or 12 months after 3MDR for a subset of participants (n=30). To investigate the possibility that some participants did not complete follow-up data collection because they did not have success with 3MDR therapy, we analyzed changes in scores Pre- vs. Post-3MDR separately for participants without follow-up (n=18) follow-up and those with data at one or more follow-up sessions (n=30). Results are presented below, in subsection Results for Analyses of Participants Without and With Follow-up Data.

**Appendix 3 - Additional Results**

**Results for Analyses of Participant Subgroups**

The main text reports results from n=48 participants. Participants were recruited from three subgroups: military members and Veterans (n=17), public safety personnel (n=21), and healthcare workers (n=21). Some participants belonged to more than one subgroup.

We analyzed changes in scores from Pre- to Post-3MDR in each of the three subgroups, presented in Appendix Table 1. Each subgroup exhibited a similar pattern of score changes as the aggregate results presented in the main text. PCL-5, PHQ-9, GAD-8, and OQ-45 questionnaire scores decreased. CD-RISC-25 resiliency scores increased. Scores derived from the CAPS-5 interview decreased. Changes were statistically significant with a few exceptions (see P-value column in Appendix Table 1).

*Appendix Table 1: Changes in Outcome Measures During the Course of 3MDR Therapy for Participant Subgroups*

| **Score** | **Pre** | **Post** | **N** | **P-value** |
| --- | --- | --- | --- | --- |
| ***Military Members and Veterans*** |  |  |  |  |
| PCL-5 | 54.9±1.8 | 40.1±4.9 | 15 | 0.02 |
| PHQ-9 | 16.9±1.4 | 11.7±1.6 | 15 | 0.01 |
| GAD-7 | 14.2±1.2 | 10.5±1.5 | 15 | 0.02 |
| OQ-45 | 101.5±5.5 | 85.1±5.3 | 14 | 0.1 |
| AUDIT | 4.3±1.3 | 4.4±1.4 | 13 | 0.9 |
| CD-RISC-25 | 53.4±4.5 | 61.6±4.3 | 13 | 0.2 |
| CAPS-5 Total Symptom Score | 50.7±2.8 | 36.8±6.2 | 9 | 0.03 |
| CAPS-5 B Re-experiencing | 13.3±0.9 | 7.9±1.7 | 9 | 0.02 |
| CAPS-5 C Avoidance | 6.1±0.6 | 4.2±1.0 | 9 | 0.04 |
| CAPS-5 D Neg Alterations | 17.7±1.4 | 12.8±2.1 | 9 | 0.07 |
| CAPS-5 E Hyperarousal | 13.4±1.0 | 11.9±1.7 | 9 | 0.4 |
| CAPS-5 Dissociation | 2.8±0.7 | 1.1±0.4 | 9 | 0.03 |
|  |  |  |  |  |
| ***Public Safety Personnel*** |  |  |  |  |
| PCL-5 | 52.1±2.5 | 37.1±4.1 | 20 | 0.0001 |
| PHQ-9 | 15.7±1.1 | 11.2±1.4 | 20 | 0.00007 |
| GAD-7 | 12.7±1.3 | 9.8±1.3 | 20 | 0.01 |
| OQ-45 | 101.2±4.0 | 85.4±4.8 | 19 | 0.00005 |
| AUDIT | 4.1±0.9 | 3.9±1.2 | 18 | 0.8 |
| CD-RISC-25 | 55.6±3.3 | 63.8±3.0 | 18 | 0.04 |
| CAPS-5 Total Symptom Score | 48.1±2.4 | 25.2±4.9 | 12 | 0.0006 |
| CAPS-5 B Re-experiencing | 12.0±0.6 | 5.5±1.4 | 12 | 0.001 |
| CAPS-5 C Avoidance | 5.8±0.4 | 2.2±0.8 | 12 | 0.002 |
| CAPS-5 D Neg Alterations | 16.6±1.0 | 9.6±1.9 | 12 | 0.003 |
| CAPS-5 E Hyperarousal | 13.7±0.9 | 7.8±1.2 | 12 | 0.0004 |
| CAPS-5 Dissociation | 2.0±0.5 | 1.2±0.5 | 12 | 0.3 |
|  |  |  |  |  |
| ***Healthcare Workers*** |  |  |  |  |
| PCL-5 | 42.2±3.7 | 22.6±3.8 | 21 | 0.00002 |
| PHQ-9 | 12.4±1.4 | 7.4±1.4 | 21 | 0.001 |
| GAD-7 | 10.4±1.3 | 5.3±1.1 | 21 | 0.0001 |
| OQ-45 | 80.0±5.7 | 65.5±7.2 | 21 | 0.006 |
| AUDIT | 3.1±0.7 | 2.1±0.4 | 21 | 0.03 |
| CD-RISC-25 | 62.2±3.0 | 69.5±3.8 | 21 | 0.005 |
| CAPS-5 Total Symptom Score | 43.9±3.6 | 20.2±4.4 | 15 | 0.0003 |
| CAPS-5 B Re-experiencing | 11.3±1.1 | 4.9±1.3 | 15 | 0.0006 |
| CAPS-5 C Avoidance | 5.7±0.5 | 2.1±0.5 | 15 | 0.0001 |
| CAPS-5 D Neg Alterations | 14.4±1.4 | 7.1±1.8 | 15 | 0.003 |
| CAPS-5 E Hyperarousal | 12.5±1.4 | 6.0±1.2 | 15 | 0.002 |
| CAPS-5 Dissociation | 4.6±0.7 | 0.9±0.5 | 15 | 0.0008 |
|  |  |  |  |  |
| **Appendix Table 1:** Statistical analysis of scores pre-3MDR (Pre) vs. post-3MDR (Post) for subgroups of participants: military members and Veterans (n=17), public safety personnel including paramedics (n=21), and healthcare workers excluding paramedics (n=21). Note that some of the n=48 participants belonged to more than one subgroup. The Post-3MDR time point is 3MDR reconsolidation session 1 for all scores except those from the CAPS-5, for which it is 3MDR reconsolidation session 2. The Pre and Post columns show the mean ± standard error for each score before and after 3MDR therapy. The N and P-value columns show, for each score, the numbers of participants contributing data and the p-values from statistical tests of the changes in mean score from pre- to post-3MDR. For additional details of participant numbers in various analyses, see Appendix subsection Details of Participant Numbers. Statistical analyses used permutation tests with 100,000 iterations. | | | | |

A subset of 30 participants completed data collection at one or more follow-up time points 3, 6, and 12 months after 3MDR. For each of the three subgroups, we analyzed changes from Pre-3MDR to Rec1/Rec2, as well as lack of change (equivalence) of scores over the follow-up time points. These analyses included participants with data at one or more follow-up sessions, as well as data at the Pre and Rec1 and/or Rec2 time points. Results from these analyses are shown in Appendix Table 2. Each of the three participant subgroups exhibited patterns of changes similar to the aggregate pattern presented in the main text. PCL-5, PHQ-9, GAD-7, OQ-45, and CAPS-5 derived scores all decreased over the course of 3MDR. Decreased scores were maintained (equivalent) through follow-up 12 months after 3MDR. These patterns were statistically significant, with a few exceptions detailed in Appendix Table 2. The military members and Veterans subgroup did not include enough participants with follow-up data to do statistical testing in this context.

*Appendix Table 2: Outcome Measures Up To 12 Months After 3MDR Therapy for Participant Subgroups*

| **Score** | **Pre** | **Rec1** | **Rec2** | **3m** | **6m** | **12m** | **N** | **P Pre-Post** | **P Equiv** |
| --- | --- | --- | --- | --- | --- | --- | --- | --- | --- |
| ***Military Members and Veterans*** |  |  |  |  |  |  |  |  |  |
| PCL-5 | 58.2±3.8 | 34.0±10.3 | 44.0±9.9 | 56.0±2.1 | 37.0±11.0 | 32.8±11.1 | 4 | - | - |
| PHQ-9 | 17.5±3.4 | 9.5±1.9 | 12.0±2.1 | 14.0±0.7 | 11.3±1.7 | 10.5±3.5 | 4 | - | - |
| GAD-7 | 16.0±3.5 | 9.5±3.2 | 13.5±1.1 | 17.0±0.7 | 10.7±2.7 | 8.0±3.5 | 4 | - | - |
| OQ-45 | 111.0±14.0 | 86.0±11.4 | 101.0±8.5 | 106.0±4.9 | 85.0±16.5 | 76.0±17.0 | 4 | - | - |
| CAPS-5 Total Symptom Score | 52.3±5.0 | - | 25.0±12.8 | 29.7±10.2 | 46.5±7.4 | - | 3 | - | - |
| CAPS-5 B Re-experiencing | 15.0±1.2 | - | 4.3±2.7 | 7.3±3.1 | 9.5±3.9 | - | 3 | - | - |
| CAPS-5 C Avoidance | 5.7±1.1 | - | 2.7±2.2 | 2.7±1.4 | 5.0±0.7 | - | 3 | - | - |
| CAPS-5 D Neg Alterations | 18.0±2.2 | - | 8.0±3.7 | 10.7±3.5 | 17.5±1.1 | - | 3 | - | - |
| CAPS-5 E Hyperarousal | 13.7±1.7 | - | 10.0±4.3 | 9.0±2.4 | 14.5±1.8 | - | 3 | - | - |
| CAPS-5 Dissociation | 3.7±1.5 | - | 1.0±0.8 | 0.7±0.5 | 3.0±2.1 | - | 3 | - | - |
|  |  |  |  |  |  |  |  |  |  |
| ***Public Safety Personnel*** |  |  |  |  |  |  |  |  |  |
| PCL-5 | 44.8±4.3 | 32.2±4.9 | 22.2±3.7 | 19.5±2.5 | 18.3±3.9 | 23.5±3.3 | 8 | 0.00001 | 0.00001 |
| PHQ-9 | 12.0±1.7 | 8.6±1.4 | 8.4±1.5 | 7.8±1.2 | 7.1±1.3 | 8.2±1.5 | 8 | 0.002 | 0.00001 |
| GAD-7 | 9.2±2.1 | 7.8±1.5 | 6.4±1.1 | 5.5±0.8 | 7.5±1.6 | 7.0±0.9 | 8 | 0.05 | 0.00006 |
| OQ-45 | 94.4±6.8 | 83.6±6.5 | 74.8±7.3 | 71.5±5.9 | 73.6±7.9 | 76.9±8.6 | 8 | 0.00004 | 0.00001 |
| CAPS-5 Tot Sympt Score | 48.0±2.0 | - | 17.6±3.3 | 17.3±3.7 | 18.6±4.4 | 23.2±5.1 | 7 | 0.02 | 0.00001 |
| CAPS-5 B Re-experiencing | 11.9±0.5 | - | 3.3±0.6 | 3.6±1.1 | 2.6±1.0 | 5.2±1.6 | 7 | 0.02 | 0.001 |
| CAPS-5 C Avoidance | 5.7±0.3 | - | 1.0±0.7 | 1.1±0.3 | 2.4±0.9 | 2.0±0.8 | 7 | 0.03 | 0.2 |
| CAPS-5 D Neg Alterations | 16.7±1.3 | - | 7.4±1.8 | 6.6±1.5 | 8.2±1.6 | 9.0±2.2 | 7 | 0.02 | 0.00001 |
| CAPS-5 E Hyperarousal | 13.7±1.0 | - | 5.9±0.9 | 6.0±1.7 | 5.4±2.0 | 7.0±1.7 | 7 | 0.02 | 0.00001 |
| CAPS-5 Dissociation | 1.4±0.7 | - | 0.9±0.5 | 0.6±0.4 | 1.0±0.6 | 0.5±0.3 | 7 | 0.8 | 0.00001 |
|  |  |  |  |  |  |  |  |  |  |
| ***Healthcare Workers*** |  |  |  |  |  |  |  |  |  |
| PCL-5 | 43.1±3.9 | 23.2±4.1 | 20.1±5.1 | 17.5±4.3 | 17.4±4.3 | 19.1±4.6 | 19 | 0.00001 | 0.00001 |
| PHQ-9 | 11.7±1.5 | 7.6±1.5 | 6.3±1.5 | 6.6±1.5 | 6.8±1.4 | 6.5±1.5 | 19 | 0.00002 | 0.00001 |
| GAD-7 | 10.4±1.4 | 5.5±1.2 | 4.9±1.2 | 4.9±1.0 | 5.2±1.1 | 4.6±1.1 | 19 | 0.00001 | 0.00001 |
| OQ-45 | 80.9±6.1 | 66.6±7.8 | 53.6±9.1 | 56.8±7.5 | 60.1±7.7 | 63.8±8.1 | 19 | 0.00007 | 0.00001 |
| CAPS-5 Tot Sympt Score | 43.9±3.6 | - | 20.2±4.4 | 19.4±4.5 | 20.1±4.5 | 23.8±4.6 | 15 | 0.0004 | 0.00001 |
| CAPS-5 B Re-experiencing | 11.3±1.1 | - | 4.9±1.3 | 5.2±1.3 | 4.9±1.2 | 4.9±1.2 | 15 | 0.0005 | 0.00001 |
| CAPS-5 C Avoidance | 5.7±0.5 | - | 2.1±0.5 | 2.0±0.6 | 2.1±0.6 | 2.8±0.7 | 15 | 0.0002 | 0.003 |
| CAPS-5 D Neg Alterations | 14.4±1.4 | - | 7.1±1.8 | 5.7±1.7 | 6.9±1.8 | 8.6±1.8 | 15 | 0.002 | 0.00001 |
| CAPS-5 E Hyperarousal | 12.5±1.4 | - | 6.0±1.2 | 6.4±1.2 | 6.3±1.3 | 7.5±1.3 | 15 | 0.002 | 0.00001 |
| CAPS-5 Dissociation | 4.6±0.7 | - | 0.9±0.5 | 1.4±0.4 | 1.1±0.4 | 1.0±0.3 | 15 | 0.0006 | 0.00001 |
|  |  |  |  |  |  |  |  |  |  |
| **Appendix Table 2:** Statistical subgroup analyses of scores collected at pre-3MDR baseline as well as reconsolidation and follow-up for subsets of participants who completed data collection at Pre, Rec2, and one or more follow-up time points. Analyses are for subgroups of participants: military members and veterans (n=17), public safety personnel including paramedics (n=21), and healthcare workers excluding paramedics (n=21). Note that some of the n=48 participants belonged to more than one subgroup. Time points include pre-3MDR baseline (Pre), 3MDR reconsolidation sessions 1 and 2 (Rec1, Rec2), and follow-up at 3, 6, and 12 months (3m, 6m, 12m). The Pre, Rec1, Rec2, 3m, 6m, and 12m columns show the mean ± standard error for each score at each time point. CAPS-5 measures were not collected at Rec1. The N column shows the numbers of participants for the pre-post analysis and equivalence test. For additional details of participant numbers in various analyses, see Appendix subsection Details of Participant Numbers. The "P Pre-Post" column shows p-values for statistical tests of the changes in mean scores from Pre to Rec2 time points. The "P Equiv" column shows p-values for statistical equivalence tests testing for a statistically significant lack of increase from Rec2 over the follow-up 3m, 6m, and 12m time points. P-values and 12 month data points are not included for military members and veterans due to insufficient sample size. Statistical analyses used permutation tests or bootstrap tests with 100,000 iterations. | | | | | | | | | |

**Results for Participants Without and With Follow-up Data**

*Appendix Table 3: Changes in Outcome Measures Over 3MDR Therapy for Participants Without and With Follow-up Data*

| **Score** | **Pre** | **Post** | **N** | **P-value** |
| --- | --- | --- | --- | --- |
| ***Participants Without Follow-up Data*** |  |  |  |  |
| PCL-5 | 53.9±2.9 | 43.2±4.9 | 16 | 0.01 |
| PHQ-9 | 17.4±1.1 | 13.4±1.5 | 16 | 0.007 |
| GAD-7 | 14.4±1.1 | 11.9±1.5 | 16 | 0.02 |
| OQ-45 | 97.9±5.1 | 88.5±5.6 | 15 | 0.05 |
| AUDIT | 3.4±1.3 | 3.5±1.3 | 14 | 1.0 |
| CD-RISC-25 | 55.6±4.0 | 60.5±3.7 | 14 | 0.3 |
| CAPS-5 | 51.4±3.2 | 43.6±4.6 | 7 | 0.08 |
| CAPS-5 B Re-experiencing | 13.0±0.9 | 10.3±1.6 | 7 | 0.09 |
| CAPS-5 C Avoidance | 6.6±0.6 | 5.1±0.8 | 7 | 0.03 |
| CAPS-5 D Neg Alterations | 18.0±1.6 | 15.3±1.6 | 7 | 0.2 |
| CAPS-5 E Hyperarousal | 13.7±1.1 | 12.9±1.0 | 7 | 0.5 |
| CAPS-5 Dissociation | 2.7±0.7 | 1.7±0.7 | 7 | 0.1 |
|  |  |  |  |  |
| ***Participants With Follow-up Data 3, 6, and/or 12 Months After 3MDR*** | | | | |
| PCL-5 | 45.7±2.9 | 26.3±3.4 | 30 | 0.0002 |
| PHQ-9 | 12.7±1.2 | 7.9±1.1 | 30 | 0.00008 |
| GAD-7 | 11.1±1.2 | 6.4±1.0 | 30 | 0.01 |
| OQ-45 | 89.0±5.2 | 71.7±5.7 | 30 | 0.00001 |
| AUDIT | 3.9±0.7 | 3.5±0.8 | 30 | 0.4 |
| CD-RISC-25 | 59.2±2.7 | 67.6±3.1 | 30 | 0.001 |
| CAPS-5 | 44.9±2.6 | 19.4±3.5 | 23 | 0.00001 |
| CAPS-5 B Re-experiencing | 11.8±0.8 | 4.4±1.0 | 23 | 0.00002 |
| CAPS-5 C Avoidance | 5.7±0.3 | 1.8±0.5 | 23 | 0.00001 |
| CAPS-5 D Neg Alterations | 14.9±1.0 | 6.8±1.3 | 23 | 0.00003 |
| CAPS-5 E Hyperarousal | 12.6±1.0 | 6.4±1.0 | 23 | 0.0001 |
| CAPS-5 Dissociation | 3.6±0.6 | 0.8±0.3 | 23 | 0.0004 |
|  |  |  |  |  |
| **Appendix Table 3:** Statistical analysis of scores pre-3MDR (Pre) vs. post-3MDR (Post) for participants without (n=18) or with (n=30) follow-up data 3, 6, and/or 12 months after 3MDR. The Post-3MDR time point is 3MDR reconsolidation session 1 for all scores except those from the CAPS-5, for which it is 3MDR reconsolidation session 2. The Pre and Post columns show the mean ± standard error for each score before and after 3MDR therapy. The N and P-value columns show, for each score, the numbers of participants contributing data and the p-values from statistical tests of the changes in mean score from pre- to post-3MDR. Statistical analyses used permutation tests with 100,000 iterations. | | | | |

**Details of Participant Numbers**

We included n=48 participants in the analyses presented in the main text. Above, we include analyses for three subgroups of participants: military and Veterans (n=17), public safety personnel (n=21), and healthcare workers (n=21), with some participants belonging to multiple subgroups.

Self-report questionnaire score data in Table 2 and Figure 1 in the main text are derived from 44-46 participants, depending on the questionnaire. Two of 48 participants did not complete questionnaire data collection from time point Rec1 onward, though they did complete the CAPS-5 interview at Rec2. These two also did not complete any follow-up data collection. For the OQ-45, AUDIT, and CD-RISC-25 scales, respectively, one, two, and two participants did not complete enough questions to generate a score at one or both time points (Pre and Rec1). These factors also account for the participant numbers in the N column in Appendix Table 1 for the self-report questionnaire scores, for the three participant subgroups, and in Appendix Table 3 for the participants without follow-up data.

All participants completed the CAPS-5 interview during 3MDR screening, but there was CAPS-5 data loss for some participants for the Pre and/or Rec2 time points. CAPS-5 data shown in Table 2 in the main text are from 30 participants for whom CAPS-5 data are available at both the Pre and Rec2 time points. Similarly, loss of CAPS-5 data for some participants accounts for the N column in Appendix Table 1 for the CAPS-5 score results and in Appendix Table 3.

A subset of participants (n=30) completed data collection at one or more follow-up time points 3, 6, and/or 12 months after 3MDR therapy. Results shown in Table 3 and Figure 2 of the main text are based on participants for whom data was available at time points Pre, Rec1 and/or Rec2, and one or more of 3m, 6m, and/or 12m. For self-report questionnaire scores, this included 29 participants, and for CAPS-5 interview scores, this included 22 participants. These same considerations account for the participant numbers for the subgroup analyses in the N column in Appendix Table 2. The military member / Veteran subgroup contributed 4 and 3 participants, respectively, for self-report questionnaire and CAPS-5 interview data. The public safety personnel subgroup contributed 8 and 7 participants, respectively. The healthcare worker subgroup included 19 and 15 participants, respectively.

**References**

Benjamini Y, Hochberg Y. (1995) Controlling the false discovery rate: a practical and powerful approach to multiple testing. *J R Stat Soc Series B*: 57(1), 289-300. DOI:[10.1111/j.2517-6161.1995.tb02031.x](https://doi.org/10.1111/j.2517-6161.1995.tb02031.x).

Bradley KA, Bush KR, Epler AJ, et al. (2003) Two brief alcohol-screening tests From the Alcohol Use Disorders Identification Test (AUDIT): Validation in a female Veterans Affairs patient population. *Arch Intern Med*: 163, 821-829. DOI:10.1001/archinte.163.7.821.

Connor KM, Davidson JRT. (2003) Development of a new resilience scale: the Connor-Davidson Resilience Scale (CD-RISC). *Depress Anxiety*: 18, 71-82. DOI:10.1002/da.10113.

Lambert MJ, Hansen NB, Umphress V, et al. (1996) *Administration and scoring manual for the Outcome Questionnaire (OQ45.2).* American Professional Credentialing Services, Wilmington, DE.

Weathers FW, Blake DD, Schnurr PP, Kaloupek DG, Marx BP, Keane TM. (2013a) *The Life Events Checklist for DSM-5 (LEC-5) – Standard*.

Weathers FW, Litz BT, Keane TM, Palmieri PA, Marx BP, Schnurr PP. (2013b) *The PTSD Checklist for DSM-5 (PCL-5) – LEC-5 and Extended Criterion A* [Measurement instrument]. Available from <http://www.ptsd.va.gov/>. Accessed November 18, 2018.
